# Egg Intake during Carbohydrate Restriction Alters Peripheral Blood Mononuclear Cell Inflammation and Cholesterol Homeostasis in Metabolic Syndrome

**DOI:** 10.3390/nu6072650

**Published:** 2014-07-18

**Authors:** Catherine J. Andersen, Ji-Young Lee, Christopher N. Blesso, Timothy P. Carr, Maria Luz Fernandez

**Affiliations:** 1 Department of Nutritional Sciences, University of Connecticut, Storrs, CT 06269, USA; E-Mails: catherine.andersen@uconn.edu (C.J.A.); ji-young.lee@uconn.edu (J.-Y.L.); christopher.blesso@uconn.edu (C.N.B.); 2 Department of Nutrition and Health Sciences, University of Nebraska, Lincoln, NE 68683, USA; E-Mail: tcarr2@unl.edu

**Keywords:** inflammation, cholesterol, peripheral blood mononuclear cells, ABCA1, eggs, metabolic syndrome

## Abstract

Egg yolk contains bioactive components that improve plasma inflammatory markers and HDL profiles in metabolic syndrome (MetS) under carbohydrate restriction. We further sought to determine whether egg yolk intake affects peripheral blood mononuclear cell (PBMC) inflammation and cholesterol homeostasis in MetS, as HDL and its associated lipid transporter ATP-binding cassette transporter A1 (ABCA1) reduce the inflammatory potential of leukocytes through modulation of cellular cholesterol content and distribution. Thirty-seven men and women classified with MetS consumed a moderate carbohydrate-restricted diet (25%–30% of energy) for 12 weeks, in addition to consuming either three whole eggs per day (EGG) or the equivalent amount of yolk-free egg substitute (SUB). Interestingly, lipopolysaccharide-induced PBMC IL-1β and TNFα secretion increased from baseline to week 12 in the SUB group only, despite increases in PBMC toll-like receptor 4 (TLR4) mRNA expression in the EGG group. Compared to baseline, ABCA1 and 3-hydroxy-3-methyl-glutaryl (HMG)-CoA reductase mRNA expression increased by week 12 in the EGG group only, whereas changes in PBMC total cholesterol positively correlated with changes in lipid raft content. Together, these findings suggest that intake of whole eggs during carbohydrate restriction alters PBMC inflammation and cholesterol homeostasis in MetS.

## 1. Introduction

Chronic, low-grade inflammation is commonly associated with obesity-related metabolic diseases, and is often indicative of organ stress and insufficient resolution of inflammatory immune responses [1,2,3]. Elevated levels of plasma inflammatory markers have been reported in cardiovascular disease (CVD), type 2 diabetes mellitus (T2DM), and metabolic syndrome (MetS) [4,5]. These cytokines, chemokines, adhesion molecules, and acute phase proteins may be derived from metabolically stressed adipose tissue, liver, and skeletal muscle, in addition to activated leukocytes [1,2,3].

Recent studies have specifically focused on the role of leukocytes in contributing to metabolic disease progression [6,7], as well as how obesity can affect basic leukocyte properties and function in conferring immunity [8]. Multiple studies have demonstrated that factors related to obesity increase the basal inflammatory profile of lymphocytes [9,10], in addition to promoting greater pro-inflammatory responses upon stimulation and activation [11,12]. The consequences of these actions can include altered monocyte and lymphocyte migration and adhesion patterns, impaired immunity, prolonged systemic inflammation, and greater difficulty in resolving acute inflammatory responses. These changes have further been linked to greater progression of atherosclerosis, arterial dysfunction, adipose tissue inflammation and dysfunction, and insulin resistance [7,11,12]. Therefore, it is important that therapeutic intervention strategies targeting markers of chronic low-grade inflammation similarly address dysregulated leukocyte signaling.

Dietary and lifestyle therapies that promote weight loss and improved diet quality have been shown to effectively ameliorate markers of systemic inflammation, including inflammatory cytokines and acute phase proteins such as C-reactive protein (CRP) and lipoprotein-associated serum amyloid A (SAA) [13,14,15]. Moderate weight loss (~5% of body weight) has additionally been shown to reduce inflammatory gene expression and nuclear factor κB (NF-κB) DNA binding activity in peripheral blood mononuclear cells (PBMC) from obese women [9]. PBMC inflammation is also differentially affected by consumption of diets rich in monounsaturated* vs.* saturated fats [16], and may be reduced by intake of antioxidant-rich foods [17].

In addition to weight loss and dietary manipulation, the regulation of leukocyte cholesterol flux has recently garnered significant attention due to its implications for inflammation, immunity, atherosclerosis, and metabolic disease [18,19,20]. Leukocytes with elevated levels of cholesterol have been shown to be more inflammatory and hyperproliferative [18,21,22], which may lead to inappropriate inflammatory hyper-responsiveness to stimuli, impaired resolution of inflammation, or misguided immune responses.

HDL-associated lipid transporters, including ATP-binding cassette transporter A1 (ABCA1) and ATP-binding cassette G1 (ABCG1), have been shown to play significant roles in the modulation of leukocyte cholesterol content. While the anti-atherogenic properties of both ABCA1 and ABCG1 have been well documented due to their capacity to participate in the initiation of reverse cholesterol transport through efflux of macrophage foam cell lipids to HDL [23,24,25], these proteins additionally possess the capacity to reduce inflammatory responsiveness through depletion of lymphocyte cholesterol content and lipid raft formation [26,27]. Cellular cholesterol depletion impairs the formation of lipid rafts within the plasma membrane where transmembrane proteins and receptors such as toll-like receptor 4 (TLR4) reside, thereby blunting the activation of inflammatory signaling [26,27,28]. ABCA1 expression in leukocytes has additionally been implicated in cellular recruitment and migration, as deletion of leukocyte-specific ABCA1 results in more advanced atherosclerotic lesions, as well as increased leukocyte counts in the liver, spleen, and peripheral blood [29]. Further, ABCA1 is thought to efflux LPS to HDL as a means of neutralizing its inflammatory effects, while also serving as a transport system for excretion of LPS from the body via the bile [30,31,32].

With previous findings coming from animal and cell studies, we set out to determine whether the dynamics between leukocyte inflammation and cholesterol flux could be altered by diet in human subjects classified with MetS—individuals at increased risk for developing CVD and T2DM [33]. We have previously demonstrated that daily consumption of whole egg during moderate carbohydrate restriction exerts favorable effects on inflammatory markers and HDL profiles in MetS. Intake of whole eggs for 12 weeks reduced plasma tumor necrosis factor α (TNFα) and SAA [13], in addition to altering HDL lipid composition and the* ex vivo* cholesterol-accepting capacity of subject serum from cholesterol-loaded macrophages [34]. These benefits were not observed in subjects consuming egg white-based, yolk-free egg substitute, suggesting that these effects are attributable to components present in egg yolk. While egg whites contain a variety of bioactive proteins with anti-microbial, anti-hypertensive, and antioxidant properties, egg yolks contain various anti-inflammatory factors, including the antioxidant carotenoids lutein and zeaxanthin, and a wide range of bioactive phospholipid species. [34,35,36]. Therefore, we sought to determine whether whole egg intake during carbohydrate restriction could further modulate inflammatory properties of PBMCs in MetS, and whether any observed changes correspond to alterations in cellular cholesterol content and distribution in lipid rafts.

## 2. Experimental Section

### 2.1. Study Design and Dietary Intervention

Men (*n* = 12) and women (*n* = 25) classified with MetS according to the National Cholesterol Education Program (NCEP): Adult Treatment Panel (ATP) III criteria [33] were recruited to participate in a 12-week parallel, randomized, single-blind intervention study. Inclusion criteria for participation included age of 30–70 years old and no history of chronic or metabolic disease. All subjects were instructed to follow a moderate carbohydrate-restricted diet (25%–30% of energy from carbohydrate, 25%–30% of energy from protein, 45%–50% of energy from fat) throughout the 12-week intervention, in addition to consuming either three whole eggs per day (EGG group, *n* = 20) or the equivalent amount of egg white-based, yolk-free egg substitute (SUB group, *n* = 17). Assignment to EGG and SUB groups was random, with subjects matched on the basis of age, gender, and body mass index. Liquid whole egg (EGG group) and egg substitute (SUB group) products (Sysco Corporation, Houston, TX, USA) were provided to subjects on a biweekly basis in coded containers. The daily serving of whole eggs provided 186 kcal, 0 g carbohydrate, 16 g protein, 12 g fat, and 534 mg cholesterol, whereas the egg substitute provided 60 kcal, 2 g carbohydrate, 14 g protein, 0 g fat, and 0 mg cholesterol. Additional details of this intervention have been previously described [13,34,35,37]. This study was approved by the Institutional Review Board at the University of Connecticut (Protocol #: H10-173).

### 2.2. Blood Collection and Body Weight

Fasting blood samples were collected at baseline and week 12 of the intervention for isolation of PBMCs and serum for cell culture assays. All samples were processed under sterile conditions. Serum was aliquoted and stored at −80 °C. Body weight was measured biweekly from baseline to week 12. As expected with moderate carbohydrate-restriction, average body weight loss in both groups was ~4% with no difference between EGG and SUB groups [13].

### 2.3. Peripheral Blood Mononuclear Cell Isolation

Fasting blood (50 mL) was collected into EDTA vacutainer tubes for isolation of PBMCs at baseline and week 12 of the intervention. Due to low blood recovery in four subjects, PBMCs were collected from 34 EGG (*n* = 18) and SUB (*n* = 15) group participants. PBMCs were isolated by density gradient centrifugation using Ficoll Paque PLUS (GE Healthcare, Pittsburgh, PA, USA) according to the manufacturer’s instructions. Whole blood was diluted with sterile PBS, layered over Ficoll Paque PLUS, and centrifuged at 400× *g* for 35 min using a Beckman Coulter centrifuge with a swing-bucket rotor to separate the PBMC buffy coat. PBMC buffy coats were collected, washed twice with PBS, and resuspended in RPMI. Aliquots of freshly isolated PBMCs were taken for collection of RNA, nuclear extracts, and whole cell lysates as described below. Remaining cells were diluted 1:1 with cryopreservation media (RPMI containing 20% fetal bovine serum, 10% dimethyl sulfoxide) and frozen at a controlled rate in CoolCell containers (BioCision, LLC, Larkspur, CA, USA) at −80 °C for at least 24 h. PBMC samples were then transferred to liquid nitrogen for long-term storage. PBMC recovery from whole blood was not sufficient to conduct every experiment on samples from each subject. Sample sizes for each measurement are reported below.

### 2.4. Quantitative Real-Time RT-PCR

PBMC mRNA expression of inflammatory genes was determined by quantitative real-time RT-PCR (qRT-PCR) [38,39]. RNA was extracted from freshly isolated PBMCs using TRIzol reagent (Invitrogen, Carlsbad, CA, USA) according to the manufacturer’s instructions. One µg of RNA was treated with DNase I (Promega, Madison, WI, USA) and reverse transcribed by MMLV reverse transcriptase (Promega, Madison, WI, USA) using a Bio-Rad C1000 Thermal Cycler (Bio-Rad, Hercules, CA, USA). qRT-PCR analysis was performed using the Sybr Green procedure with a Bio-Rad CFX96 system (Bio-Rad, Hercules, CA, USA). Primer sequences were designed according to the GenBank database, and are presented in Table 1. Expression of mRNA values was calculated using the threshold cycle (*C*_t_) value. Relative expression levels of each target gene were calculated using the comparative 2^−ΔΔ*C*t^ method following normalization to 18S rRNA expression [38,39,40].

**Table 1:** Quantitative real-time RT-PCR primer sequences.

Gene	Forward Primer	Reverse Primer
ABCA1	5′-TTTCTCAGACAACACTTGACCAAGTA-3′	5′-GGTTTTTGTGTAATGAGAGGTCTTTTAA-3′
ABCG1	5′-CGGAGGGCAGCTGTGAAC-3′	5′-GGGTCCTTCAGGAACCGAAT-3′
HMGCR	5′-CCCAGTTGTGCGTCTTCCA-3′	5′-TTCGAGCCAGGCTTTCACTT-3′
IL-1β	5′-ACGATGCACCTGTACGATCACT-3′	5′-CACCAAGCTTTTTTGCTGTGAGT-3′
IL-6	5′-GCTGCAGGCACAGAACCA-3′	5′-GCTGCGCAGAATGAGATGAG-3′
LDLR	5′-ACTGGGTTGACTCCAAACTTCAC-3′	5′-GGTTGCCCCCGTTGACA-3′
TLR4	5′-GCAGGTGCTGGATTTATC-3′	5′-GTAGAGAGGTGGCTTAGG-3′
TNFα	5′-GGGACCTCTCTCTAATCA-3′	5′-CTACAACATGGGCTACAG-3′
18S RNA	5′-CGGCTACCACATCCAAGGAA-3′	5′-GCTGGAATTACCGCGGCT-3′

### 2.5. NF-κB p65 DNA Binding Activity

Nuclear fractions were collected from freshly isolated PBMCs using a Nuclear Extraction kit (Active Motif, Carlsbad, CA, USA) following the manufacturer’s instructions [38,41]. Nuclear fractions were stored at −80 °C until analysis. Total nuclear protein was determined by BCA assay (Pierce, Rockford, IL, USA), then used to determine the DNA binding activity of the NF-κB p65 subunit using the TransAM^®^ NF-κB p65 Kit (Active Motif, Carlsbad, CA, USA) according to the manufacturer’s instructions. Data are presented as the spectrophotometric reading at optical density (OD) 450 nm.

### 2.6. PBMC Stimulation Assays

Inflammatory responses to LPS were assessed in a subset of subject PBMCs (EGG: *n* = 5; SUB: *n* = 5). PBMCs were rapidly thawed in a 37 °C water bath, then quantified and assessed for viability using an automated cell counter (Bio-Rad, Hercules, CA, USA) with trypan blue exclusion. Maintenance of viability was further confirmed in cultured PBMCs. 2 × 10^6^ viable cells/mL/well were plated in 12-well plates, then treated with or without lipopolysaccharide (LPS; 500 ng/mL) for 6 hours at 37 °C in serum-free RPMI. Media was then collected and assayed for TNFα (BD Biosciences, San Jose, CA, USA), IL-1β, and IL-10 (Abcam, Cambridge, MA, USA) by ELISA according to the manufacturer’s instructions.

### 2.7. Quantification of PBMC Cholesterol Content

PBMC cholesterol content (EGG: *n =* 15; SUB: *n =* 13) was measured by gas chromatography/mass spectrometry (GC/MS) using an Agilent 7890 GC/MS equipped with an Agilent HP-5MS capillary column with dimensions: 30 m × 0.25 mm (0.25 μm film thickness). PBMC cholesterol was extracted by isopropyl alcohol following the addition of 5α-cholestane as an internal standard. PBMC lipids were dissolved in hexane prior to injection. Run conditions were: Initial temperature = 150 °C; Temp ramp = 15 °C/min to 225 °C (hold for 5 min); Temp ramp = 15 °C/min to 300 °C (hold for 10 min); with a total run time of 25 min. Helium was used as the carrier gas. Cholesterol values were normalized to total cell protein as determined by BCA assay (Pierce, Rockford, IL, USA).

### 2.8. Quantification of PBMC ABCA1 Protein

Whole cell lysates were collected from freshly isolated PBMCs (EGG: *n* = 11; SUB: *n* = 10) as described by Rasmussen *et al.* [39]. Briefly, following isolation, PBMCs were pelleted and resuspended in whole cell lysis buffer (150 mmol/L NaCl, 25 mmol/L Tris-HCL, pH 7.4, 1% Triton X-100) containing Protease Inhibitor Cocktail set III (Calbiochem, Darmstadt, Germany) for 20 min on ice. Cells were repelleted to collect the supernatant for storage at −80 °C until analysis. ABCA1 protein content was determined by ELISA (BIOTANG Inc., Waltham, MA, USA). Data are presented as ng protein per 1 × 10^6^ cells.

### 2.9. Lipid Raft Staining, Microscopy, and Quantification

Lipid rafts were quantified using the Vybrant Alexa Fluor 488 Lipid Raft Labeling Kit (Molecular Probes, Eugene, OR, USA) according to the manufacturer’s instructions. Briefly, cryopreserved PBMCs were rapidly thawed at 37 °C, quantified and assessed for viability. 1 × 10^6^ viable cells were then labeled with a fluorescent cholera toxin subunit B (CT-B)-Alexa Fluor 488 conjugate, which binds to the pentasaccharide chain of plasma membrane ganglioside G_M1_ localized within lipid raft domains. It has previously been shown that leukocyte lipids rafts are maintained following freezing [42]. An anti-CT-B antibody was then added to induce crosslinking of the fluorescent conjugate. PBMCs were loaded onto chamber slides to visualize lipid raft fluorescence using a Leica TCS SP2 Laser Scanning Confocal microscope (Leica Microsystems Inc., Buffalo Grove, IL, USA) and 40X oil objective. Total lipid raft fluorescence was quantified using a fluorescence plate reader at excitation 488 and absorbance/emission 495/519, then normalized to cell protein as determined by BCA assay (Pierce, Rockford, IL, USA).

### 2.10. Statistical Analysis

All statistical analyses were performed using SPSS version 18. Paired *t* tests were used to test differences between baseline* vs.* week 12 values within EGG or SUB groups. Independent *t* tests were used to compare the differences in absolute or percent change in variables between groups. Bivariate Pearson correlations were used to determine relationships between parameters. Data are reported as mean ± SEM unless noted otherwise. *P* < 0.05 was considered significant.

## 3. Results

### 3.1. Effects of Egg Intake during Moderate Carbohydrate Restriction on Inflammatory Gene Expression

**Figure 1:**
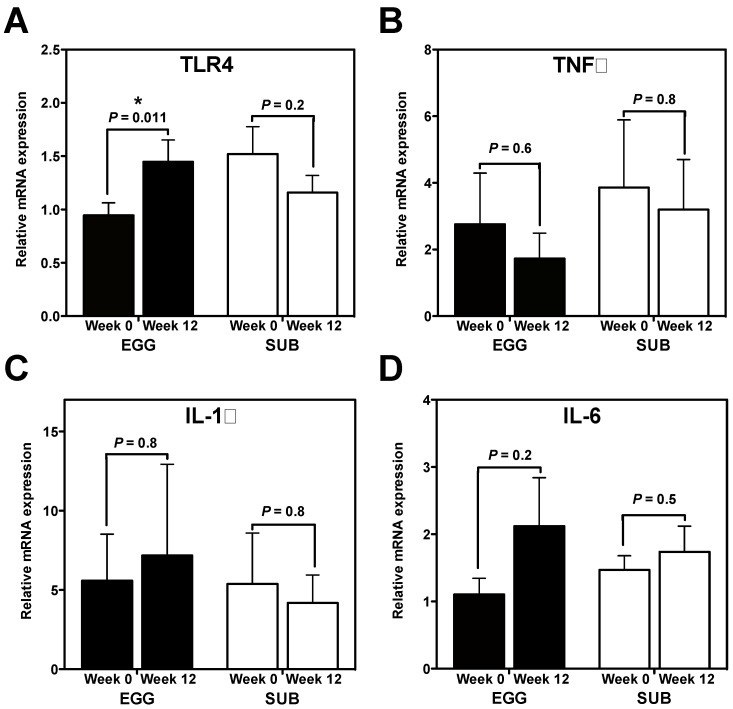
Whole egg intake during moderate carbohydrate restriction increases PBMC mRNA expression of TLR4. PBMC mRNA expression of pro-inflammatory genes at baseline and week 12 of a moderate carbohydrate-restricted diet plus whole egg (*n* = 18) or egg substitute (*n* = 15) intake. qRT-PCR was used to measure PBMC mRNA expression of (**A**) TLR4; (**B**) TNFα; (**C**) IL-1β; and (**D**) IL-6. All data were normalized to 18S rRNA expression and expressed as mean ± SEM. * *P* = 0.011, paired *t* test comparing PBMC TLR4 mRNA expression between baseline and week 12 in the EGG group.

We sought to determine whether our dietary intervention modulated inflammatory PBMC gene expression. Interestingly, while we did not observe changes in IL-6 (Figure 1B), IL-1β (Figure 1C), or TNFα (Figure 1D) mRNA expression in either group, TLR4 mRNA expression increased from baseline to week 12 in the EGG group only (Figure 1A). No significant changes in PBMC NF-κB p65 DNA binding activity were observed in either group (data not shown).

### 3.2. Inflammatory Challenge

**Figure 2:**
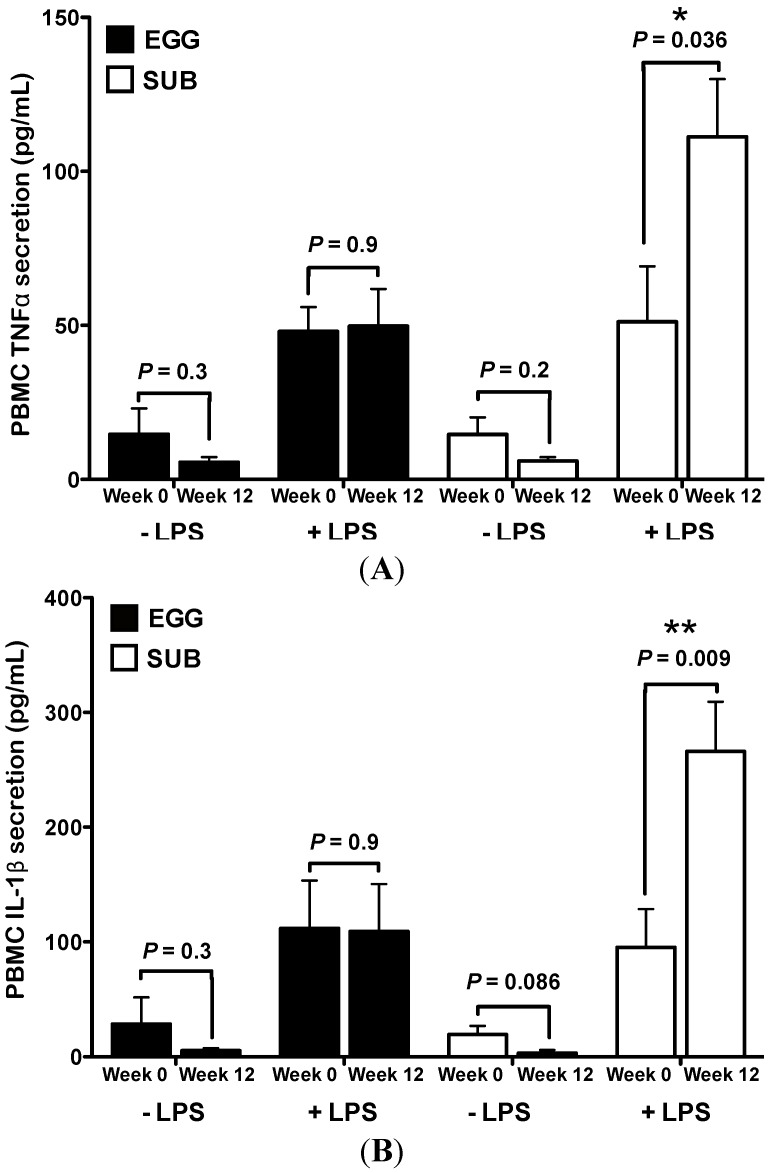
Egg substitute intake during carbohydrate restriction increases PBMC inflammatory responsiveness to LPS. Whole egg (*n* = 5) and egg substitute (*n* = 5) intake during carbohydrate restriction differentially affected LPS-induced PBMC cytokine secretion. (**A**) ELISA analysis for PBMC TNFα and (**B**) IL-1β secretion with or without stimulation with LPS for 6 h at baseline and week 12 of the dietary intervention. Data are represented as mean ± SEM. * *P* < 0.05, ** *P* < 0.01; paired *t* test comparing the difference between baseline *vs.* week 12 in SUB group.

We further assessed the inflammatory potential of PBMCs following egg intake during moderate carbohydrate restriction by measuring TNFα, IL-1β, and IL-10 secretion in response to LPS stimuli. Under basal, non-stimulated conditions, no significant changes in PBMC TNFα (Figure 2A) or IL-1β (Figure 2B) production from baseline to week 12 were observed in either group. Conversely, LPS-induced PBMC production of TNFα and IL-1β were increased from baseline to week 12 in the SUB group, whereas no changes in TNFα or IL-1β were observed in the EGG group (Figure 2A,B). No significant differences in IL-10 levels were observed under LPS or non-stimulated conditions (data not shown).

### 3.3. Cholesterol Gene Expression

**Figure 3:**
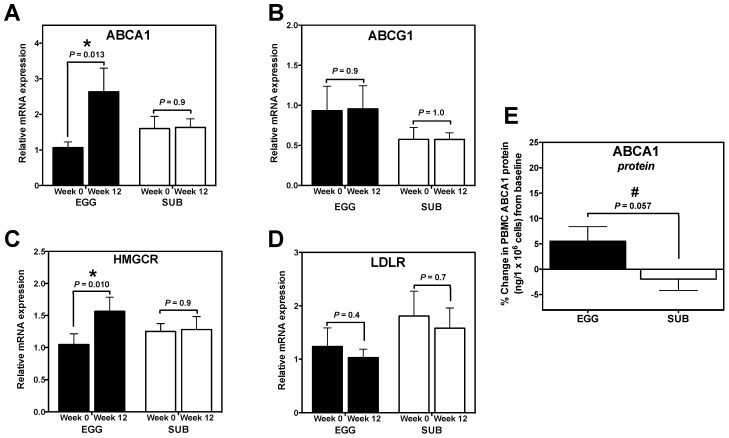
Egg intake during carbohydrate restriction alters PBMC cholesterol gene expression. PBMC expression of cholesterol genes at baseline and week 12 of a moderate carbohydrate-restricted diet plus whole egg (*n* = 18) or egg substitute (*n* = 15) intake. qRT-PCR was used to measure PBMC mRNA expression of (**A**) ABCA1; (**B**) ABCG1; (**C**) HMGCR; and (**D**) LDLR. qRT-PCR data were normalized to 18S rRNA expression. Differences between baseline and week 12 determined by paired *t* test; (**E**) ABCA1 protein was quantified in whole cell lysates collected from freshly isolated PBMCs (EGG: *n* = 11; SUB: *n* = 10) at baseline and week 12 of the intervention. Data are represented as % change in ABCA1 (ng/1 × 10^6^ cells) from baseline to week 12. * *P* < 0.05; paired *t* test comparing the difference between baseline* vs.* week 12 in EGG group.^ #^ *P* = 0.057; independent *t* test comparing the difference in % change in ABCA1 protein expression between EGG and SUB groups.

We further aimed to identify whether this change corresponded to alterations in the expression of genes related to cholesterol efflux (ABCA1 and ABCG1), synthesis (HMGCR), and uptake (LDLR). Interestingly, ABCA1 mRNA expression significantly increased from baseline to week 12 in the EGG group only (Figure 3A). We similarly observed a trend toward an increase in ABCA1 protein in the EGG group from baseline to week 12 (*P* = 0.07; data not shown), as well as a strong trend toward greater percent change in ABCA1 protein from baseline in the EGG group (+5.5% ± 9.4%) when compared to the SUB group (−1.9% ± 7.1%) (Figure 3E). EGG group-specific increases in HMGCR mRNA expression were also observed from baseline to week 12 (Figure 3C), whereas no changes in ABCG1 (Figure 3B) or LDLR mRNA expression were observed in either group (Figure 3D).

### 3.4. Effects of Egg Intake on PBMC Cholesterol and Lipid Raft Content

**Figure 4:**
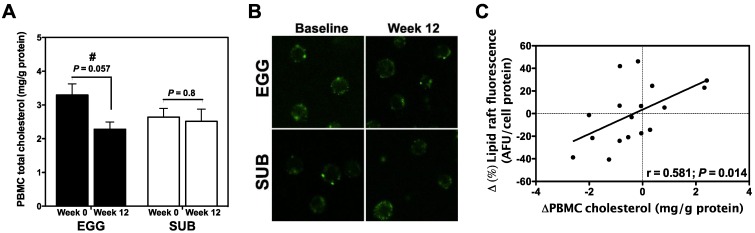
Effects of egg intake and carbohydrate restriction on PBMC cholesterol and lipid raft content. (**A**) PBMC cholesterol was measured by GC/MS and normalized to cell protein. Values are presented as mean ± SEM. ^#^ *P* = 0.057 for paired *t* test between baseline and week 12 in the EGG group. EGG: *n =* 15; SUB: *n =* 13; (**B**) PBMC lipid rafts were labeled with the Vybrant Alexa Fluor 488 Lipid Raft Labeling Kit (Molecular Probes, Eugene, OR, USA) and visualized using a Leica TCS SP2 Laser Scanning Confocal microscope (Leica Microsystems Inc., Buffalo Grove, IL, USA), 40× oil objective; (**C**) Relationship between percent changes in total lipid raft fluorescence (quantified by fluorescence plate reader) and PBMC cholesterol content as determined by Pearson correlation.

Total PBMC cholesterol content was measured to determine whether egg consumption altered leukocyte cholesterol levels. There was a strong trend towards a decrease in PBMC cholesterol content from baseline to week 12 in the EGG group (*P* = 0.057), whereas no changes in cellular cholesterol levels were observed in the SUB group (Figure 4A). No difference in baseline PBMC cholesterol content was observed between groups (*P* = 0.14; data not shown). We further sought to determine whether our intervention altered the cellular distribution of cholesterol and lipid raft formation. Lipid rafts were labeled with the Vybrant Alexa Fluor 488 Lipid Raft Labeling Kit and visualized using a Leica TCS SP2 Laser Scanning Confocal microscope (Figure 4B). Total lipid raft fluorescence was quantified using a fluorescence plate reader, and revealed no changes in lipid raft formation from baseline to week 12 in either EGG or SUB group (data not shown). However, we found that changes in PBMC total cholesterol positively correlated with percent changes in lipid raft content (Figure 4C).

## 4. Discussion

Elevated levels of basal leukocyte inflammation have been demonstrated in obesity and metabolic syndrome [9,10,43], and have been implicated in leukocyte-driven progression of metabolic disease [6,7]. HDL-leukocyte interactions via ABCA1 further modify cellular cholesterol flux and inflammatory potential; therefore, dietary strategies that alter HDL-leukocyte dynamics may have profound effects on obesity-related disease progression. Since we have previously demonstrated that bioactive nutrient-rich egg yolk improves plasma inflammatory markers and HDL profiles in metabolic syndrome (MetS) under carbohydrate restriction, we further sought to determine whether egg yolk intake affects peripheral blood mononuclear cell (PBMC) inflammation and cholesterol homeostasis in MetS. Interestingly, whole egg intake during carbohydrate restriction did not alter inflammatory PBMC responsiveness to LPS, whereas LPS-induced PBMC TNFα and IL-1β secretion was increased by moderate carbohydrate restriction and egg substitute intake. These differences were observed despite increases in PBMC TLR4 mRNA expression in the whole egg group. We additionally detected significant increases in PBMC ABCA1 and HMGCR mRNA expression from whole egg intake only, in addition to a trend toward greater increases ABCA1 protein. Further, we observed a positive correlation between changes in PBMC cholesterol and lipid raft content. Together, these findings suggest that PBMC inflammation and cholesterol homeostasis can be modulated by egg intake and moderate carbohydrate restriction in MetS.

Previous studies have reported reductions in inflammatory gene expression in PBMCs following moderate weight loss [9]. This phenomenon is thought to be indicative of global improvements in metabolic tissue stress and function, thereby reducing the presence of pathogenic factors capable of activating leukocytes, while also promoting the resolution of inflammatory responses [1,7,9]. However, despite moderate weight loss and decreases in plasma TNFα and SAA from whole egg consumption in this population [13], we did not observe reductions in PBMC mRNA expression of TNFα, IL-1β, or IL-6 from baseline to week 12. Conversely, whole egg intake increased PBMC mRNA expression of TLR4—a member of the TLR family of pattern recognition receptors that recognizes exogenous and endogenous ligands, including Gram-negative bacteria-derived LPS and fatty acids [44]. Although the subjects in this study reduced body weight by an average of ~4% [13], it is possible that changes in body weight were not significant enough to alter inflammatory gene expression in PBMCs. It is also possible that the moderate carbohydrate-restricted diet, which provided 45%–50% of energy from fat [13], blunted any changes in inflammatory gene expression associated with weight loss. High-fat diets have been associated with increased PBMC mRNA expression of IL-8 [45], higher nuclear NF-κB p65 protein levels [16], and greater TLR4 expression in a variety of tissues, including the intestine [46] and adipose tissue [47]. While dietary fat intake did not differ between EGG and SUB groups as previously reported [13], it is possible that the fatty acids provided by whole eggs were more bioavailable due to the abundance of yolk phospholipids [34,48,49], which may have contributed to the EGG group-specific increases in TLR4 mRNA expression in PBMCs.

Despite observed changes in TLR4 gene expression, our study is limited in that only mRNA levels of TLR4 were measured due to a lack of available sample to assess protein levels. While various inflammatory factors differentially regulate TLR4 mRNA expression, functional activity of this receptor is further regulated at the level of protein translation, cell surface localization, accessory molecule availability and expression, and plasma membrane composition [50]. Interestingly, omega-3 fatty acids have been shown to inhibit LPS- or saturated fatty acid-induced TLR4 activation by altering plasma membrane lipid raft composition, thereby preventing the assembly of TLR4 homodimers and signaling component complexes that is critical for inflammatory signal transduction [51]. Similar impairments in TLR4 signaling have been demonstrated via depletion of cellular cholesterol—an important structural component of lipid rafts [22,27]. Therefore, simply observing changes in TLR4 mRNA expression does not necessarily signify changes in cellular inflammatory potential.

Accordingly, daily whole egg intake for 12 weeks rendered PBMCs less responsive to LPS-induced cytokine secretion when compared to responses from egg substitute consumption during carbohydrate restriction. These findings suggest that (1) carbohydrate restriction (in conjunction with egg substitute intake) induced greater inflammatory responses upon PBMC stimulation with LPS; and (2) a component of the egg yolk blunted increased PBMC responsiveness to LPS induced by carbohydrate restriction. Egg yolks contain various anti-inflammatory factors, including the antioxidant carotenoids lutein and zeaxanthin, and numerous bioactive phospholipid species [34,35,36]. Additionally, given the changes in HDL composition and the increased cholesterol-accepting capacity of serum from egg intake described in previous studies [34,52], we hypothesized that the differences in PBMC inflammatory potential between the EGG and SUB groups could be due to changes in cellular cholesterol content, lipid raft formation, and ABCA1 expression. TLR4 signaling is dependent upon plasma membrane composition and lipid raft integrity to facilitate the convergence of LPS receptor complex components required for LPS/TLR4-mediated inflammatory signaling [50,53]. The inflammatory potential of leukocytes is further regulated through modulation of the HDL-associated lipid transporter ABCA1, where greater expression of these membrane transporters is known to deplete lipid raft cholesterol and suppress TLR4-mediated inflammatory signaling [22,27]. Therefore, it is possible that the effects of egg intake during carbohydrate restriction on PBMC inflammatory potential may be attributable to alterations in HDL-leukocyte dynamics.

Correspondingly, we observed that egg consumption trended toward decreasing PBMC cholesterol content after 12 weeks with concomitant increases in ABCA1 expression. In addition to EGG group-specific increases in ABCA1 mRNA expression, we additionally observed increased PBMC mRNA expression of HMGCR in the EGG group only, whereas no changes in ABCG1 or LDLR were observed in either EGG or SUB group. While the change in PBMC cholesterol content in the EGG group did not reach significance (*P* = 0.057), slight shifts in cellular content or compartmentalization may explain the observed increase in HMGCR mRNA expression, traditionally driven by sterol regulatory element binding protein-2 (SREBP2)-mediated activation in response to reduced cellular cholesterol levels [54].

Increased ABCA1 mRNA expression observed in this study may be due to the improved metabolic and inflammatory milieu from egg intake during moderate carbohydrate restriction. ABCA1 expression is reduced in patients with obesity-related metabolic diseases such as hypertension and T2DM [55,56], mouse models of insulin resistance and diabetes [57], and by pro-inflammatory mediators such as TNFα and CRP [58,59]. We have previously demonstrated that whole egg intake during moderate carbohydrate restriction reduces TNFα and SAA in this same MetS population [13], whereas no changes were observed in the group consuming egg substitute. Further, insulin resistance (HOMA-IR) was reduced to a greater extent in subjects consuming whole eggs [37]. Together, these findings suggest that whole egg intake during moderate carbohydrate restriction promotes global metabolic improvements that favors ABCA1 expression.

ABCA1 has repeatedly been shown to serve as a link between cellular cholesterol flux and inflammatory potential. Landry* et al.* [60] demonstrated that ABCA1 expression leads to significant redistribution of cholesterol and sphingomyelin from lipid rafts to non-raft regions of cell membranes through its ATPase-related and efflux functions [60]. Cholesterol serves as an essential structural component of lipid rafts, which are dynamic cholesterol-rich microdomains within the exoplasmic leaflets of the phospholipid bilayer of plasma membranes where transmembrane proteins and receptors reside—including pattern recognition receptors such as TLR4 [28]. Elevated levels of cellular cholesterol favor the formation of lipid rafts, and have been associated with increased pro-inflammatory responses in macrophages and T lymphocytes due to lowered cellular activation thresholds [18,21,22]. ABCA1-mediated reductions in lipid raft content have been shown to increase ADAM17-mediated cleavage of TNF and TNF receptors, which may result in reduced TNFα signaling [61]. Lipid raft structure also affects TLR signaling, as TLR4 and MyD88/TRIF-mediated inflammatory gene expression was significantly increased in peritoneal macrophages isolated from *ABCA1*^−/−^, *ABCG1*^−/−^, and *ABCA1*^−/−^*ABCG1*^−/−^ mice [19]. ApoA-I-ABCA1 interactions have also been shown to trigger JAK2-mediated activation of STAT3, which can suppress LPS-induced pro-inflammatory gene expression of TNFα and IL-6 in macrophages [62,63]. Interestingly, changes in PBMC cholesterol levels between baseline and week 12 positively correlated with percent changes in PBMC lipid raft content. This is the first study to our knowledge that has investigated the effects of diet on leukocyte lipid rafts within the context of human intervention trials. Together, these findings further support our observations that egg intake concomitantly increases ABCA1 expression while blunting the increases in LPS-induced cytokine secretion from carbohydrate restriction.

## 5. Conclusions

We have demonstrated that whole egg intake during moderate carbohydrate restriction alters PBMC inflammation and cholesterol homeostasis. The data presented in this study highlight a novel perspective on the anti-inflammatory and lipid-modulating properties of this dietary intervention. Given the significant role of leukocytes in immunity and chronic disease, regulation of cellular cholesterol flux and lipid raft formation may have important implications for the physiological consequences of obesity. Therefore, our findings support the need for future studies to determine the clinical consequences of our observations in relation to metabolic disease progression and immunity.
